# Differences in soil nutrient and microbial characteristics between invasive *Ageratina adenophora* and native plant communities

**DOI:** 10.1371/journal.pone.0325193

**Published:** 2025-06-13

**Authors:** Tian Wu, Qinghong Duan, Hongbin Zhang, Weiming Xiu, Zhilin Jiang

**Affiliations:** 1 Yunnan Key Laboratory of Plateau Geographic Processes and Environmental Change, Faculty of Geographical Science, Yunnan Normal University, Kunming, China; 2 Pu’er Green Economy Development Research Institute, Pu’er University, Pu’er, China; 3 Rural Energy and Environment Agency, Ministry of Agriculture and Rural Affairs, Beijing, China; 4 Agro-Environmental Protection Institute, Ministry of Agriculture and Rural Affairs, Tianjin, China; Universidade de Coimbra, PORTUGAL

## Abstract

Much emphasis has been placed on the negative consequences of alien species on resident ecosystems. Nevertheless, quantitative studies on the effects of invasive and native plant communities on soil nutrients and microbial features were rarely conducted. This study investigated soil microbes and soil nutrients associated with different degrees of *Ageratina adenophora* invasion and plant communities (Gramineae and Dicotyledons). The correlations between soil microbes and soil nutrients were analyzed. The findings indicated that the invasion of *A. adenophora* markedly elevated the levels of soil total nitrogen (TN), nitrate nitrogen (NO_3_^-^-N), ammonium nitrogen (NH_4_^+^-N), available potassium (AK) and available phosphorus (AP), while decreasing the concentrations of soil total phosphorus (TP) and total potassium (TK) in comparison with those in non-invaded areas. The concentrations of NO_3_^-^-N, NH_4_^+^-N, and AK in strongly invasive plant communities were significantly greater than those in the Gramineae and Dicotyledon groups. The soil microbial richness of the *A. adenophora* invasive plant community was higher than that of the native plant community. In contrast, the soil microbial evenness was lower than that of the native plant community, but the soil microbial dominance increased with the increasing degree of *A. adenophora* invasion. Meanwhile, the number of soil bacteria, fungi, actinomycetes, nitrogen-fixing bacteria, potassium-solubilizing bacteria and phosphate-solubilizing bacteria in the invasive plant community increased significantly with increasing degree of *A. adenophora* invasion. The soil microbial richness of the invasive plant community dominated by *A. adenophora* was higher than that of the native plant community. In contrast, soil microbial evenness was reduced in the invasive community compared to the native plant community, while microbial dominance increased with the extent of *A. adenophora* invasion. The number of bacteria, fungi, actinomycetes, nitrogen-fixing bacteria, potassium-solubilizing bacteria and phosphate-solubilizing bacteria in the soil of the invasive plant community increased significantly. Furthermore, the variation trend of the Simpson diversity index (D) was that of the Shannon diversity index (H) of the soil microbes in this study. The McIntosh diversity index (U) showed a consistent decrease with the increasing degree of *A. adenophora* invasion. Moreover, after the correlation coefficients between soil microbes and nutrients were analyzed, we found that there was a significant positive correlation between soil nutrients and microbial richness in both the *A. adenophora* invasive community and the native plant community. Compared with plant communities, *A. adenophora* invasion can greatly alter the soil nutrient and microbial characteristics and the trade-offs of soil nutrients supply and demand, which may facilitate growth. The soil microbial diversity in different communities may be important factors that led to changes of soil nutrients. *A. adenophora* altered the trade-offs of soil nutrients supply and demand by changing the composition and diversity of soil microbes, which may be a critical ecological mechanism of the successful invasion of the exotic weed *A. adenophora* successful invasion.

## 1. Introduction

Invasive species and ecosystems have been the focus of ecological research [1,2]. The success of exotic species invasions and the effectiveness of ecosystem resistance are intrinsically linked to their respective characteristics [3,4]. Moreover, interspecific competition, ecological interactions (e.g., competition, predation and symbiosis), and environmental changes influence both the success rate of invasive species and the resilience of ecosystems, thereby shaping the dynamic equilibrium between them [5,6]. The invasion of exotic species significantly impacts on the flow of matter and energy, thereby influencing the structure and function of the ecosystem [7]. In soil ecosystems, microbes serve as crucial and active biological factors that directly participated in processes such as plant litter decomposition, nutrient cycling and root nutrient absorption. They have significant effects on plant growth, interspecific competition and the structure and function of ecosystems [8,9]. These invasive plants can alter the physical and chemical properties of the soil through root exudates and litter decomposition, which further affects the soil microbial community within the invaded ecosystem. It is obvious that these alterations not only affect the availability of soil nutrients but also redefine competitive relationships among plants, as different plant species have different requirements and utilizations for soil nutrients [10]. Furthermore, invasive plants can smartly change the soil microbial and the nutrient composition structure of the invaded ecosystem community to favor their absorption and utilization. They can grow faster than native plants, thus competing and excluding native plant species in the invaded ecosystem and becoming the dominant species [11,12]. The successful invasion of exotic plants threatens the species composition and biodiversity of the ecosystem, thereby affecting its stability and ecological function.

The Crofton weed, *Ageratina adenophora* (Sprengel) R. King & H. Robinson (Synonym: *Eupatorium adenophorum* Sprengel), which originated in Mexico and Costa Rica, is an invasive species in China. As a perennial herbaceous species, it has invaded more than 30 countries and regions of tropical and subtropical zones [13,14]. In the 1940s, it spread from Burma into the south Lincang (e.g., Cangyuan and Gengma) of Yunnan Province in China. *A. adenophora* has subsequently widely spread widely throughout southwestern China, including Yunnan, Guizhou, Sichuan, Guangxi, Xizang Provinces and Chongqing, where it continues to spread eastward and northward at a speed of 20 km per year [15–17]. *A. adenophora* has caused severe economic losses in agriculture, forestry and livestock, severely damaging the ecology and environment of the native habitat of China. To understand the mechanism of the invasion of *A. adenophora* and to prevent its further expansion, many researchers have carried out substantial amount of work. Many studies showed that a high level of genetic diversity was detected in *A. adenophora*, which may increase its adaptability to various habitats [18] and exhibit morphological and structural plasticity [19]. Some findings have confirmed that allelochemicals from *A. adenophora* have severe negative effects on the native receptor plants (such as upland rice), whose growth and development includ mortality within a short period [20]. More research found that *A. adenophora* invasion alters underground microbial communities in invaded areas and modifies the soil biota to facilitate its continued invasion, which is a self-reinforcing invasion mechanism [18]. Some research has shown that habitats with high diversity and complexity strongly resisted the *A. adenophora* invasion, whereas disturbed habitats favored invasion. For example, the impacts of various plant functional communities, including annual and perennial Gramineae plants and their mixtures communities, demonstrated that *A. adenophora* seeds germinated rapidly in non-Gramineae community but were inhibited in Gramineae community, with mixed plant communities exerting a stronger inhibitory effect on seed germination and seedling survival compared to monocultures [21]. While numerous studies have examined the invasion mechanisms and control strategies of *A. adenophora*, research on the subsurface characteristics of plant communities established through its successful invasion, as well as those of native plant community remains sparse. These subterranean traits provide the most direct and reliable evidence for understanding the ecological mechanisms and resistance of *A. adenophora*.

In this study, our research question: Do invasive species and native plants differently affect soil nutrients and microbial diversity? If so, what is the extent of this impact, and what interactions exist between them? To assess the effects of the invasive plant *A. adenophora* and different native plants on community soil nutrients and microbial diversity, the contents of soil nutrients and different other soil microbial contents were measured across multiple representative plots. To compare the effects of the invasive plant *A. adenophora* and native plants on soil characteristics, soil microbial diversity indices were evaluated. Differences in soil nutrients and microbial characteristics between the invasive plant *A. adenophora* and native plant communities were then quantified.

## 2. Materials and methods

### 2.1 Experimental site

The experimental site is located near Qilin Mountain in Chengjiang County, Yunnan Province, China (N: 24°42′, E: 102°52′), at an elevation of 1957−2015 m, with a subtropical plateau monsoon climate. The annual rainfall is 900−1200 mm, with an uneven distribution between months and distinct dry and wet seasons. The interval from mid-May to mid-October constitutes approximately 85% of the annual precipitation, referred to as the rainy season. In contrast, the duration from late October to early May of the subsequent year experiences comparatively reduced rainfall, termed the dry season. The average annual temperature is approximately 16.8°C, with extreme maximum and minimum temperatures of 33.7°C and −3.9°C, respectively. The soil type is representative of the red soil, which is the dominant soil type in central Yunnan and is widely distributed across southern China. *Artemisia argyi*, *Oxalis corniculata*, *Clinopodium confine*, *Stellaria chinensis*, *Chenopodium album*, *Agrimonia pilosa*, *Themeda triandra*, *Imperata cylindrica*, *Cymbopogon gesoringii*, *Arthraxon hispidus*, *Eragrostis pilosa*, *Eleusine indica*, *Setaria faberii* and *Digitaria sanguinalis* are the native herbaceous plants in the area. *A. adenophora* is the main invasive plant species.

### 2.2 Experimental design and soil sample collection

#### 2.2.1 Experimental design.

Based on the impact of *A. adenophora* invasion on native plant communities, four types of plant communities were chosen as experimental sites. These included a heavily invasive community (HIC), a lightly invasive community (LIC) and two native plant communities: the Gramineae community (NGC) and the Dicotyledon community (NDC). The vegetation coverage and species information for each plant community are presented in Table 1. We interviewed native environmental protection managers and residents to understand the approximate time of *A. adenophora* invasion. The image projection method was used to measure and calculate the coverage of *A. adenophora* and its native species

**Table 1:** Four plant communities with different invasions of *A. adenophora* in the experimental design.

Main characteristics	Plant community			
	HIC	LIC	NGC	NDC
Time of invasion	≥10 years	About 3 years	0	0
*A. adenophora* coverage	≥60%	10% ~ 30%	0%	0%
Native plant coverage	≤10%	30% ~ 50%	≥70%	≥70%
Native plant species composition	*Artemisia argyi*, *Imperata cylindrica*, *Chrysopogon zizanioides*	*Artemisia argyi*, *Imperata cylindrica*, *Chrysopogon zizanioides*, *Thysanolaena latifolia*, *Eragrostis pilosa*	*Thysanolaena latifolia*, *Imperata cylindrica, Chrysopogon zizanioides*, *Arthraxon hispidus, Eragrostis pilosa*, *Eleusine indica*, *Setaria faberii*, *Digitaria sanguinalis*	*Artemisia argyi*, *Agrimonia pilosa*, *Oxalis corniculata*, *Clinopodium polycephalum*, *Stellaria media*

#### 2.2.2 Soil sample collection and preservation analysis.

**Fig 1:**
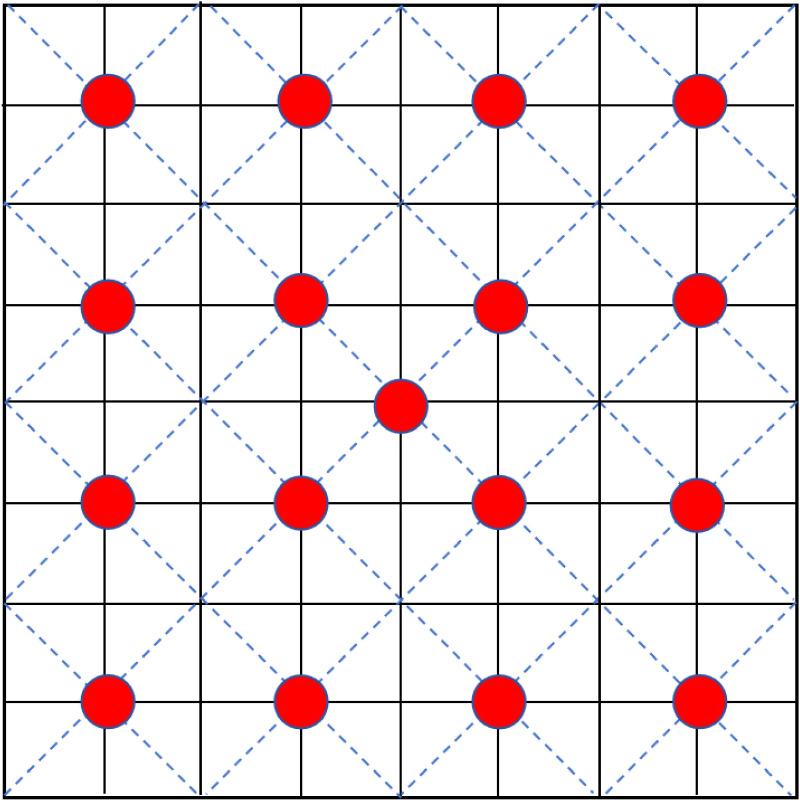
Example diagram of the soil collection sites of invasive *A. adenophora* and native plants. The diagonal center sampling method is adopted, and the red circles indicate the sampling points, for a total of 17.

Five sites were randomly selected from the four aforementioned plant communities in each plot. Each community was replicated five times, with each site covering a standard area of 4 m × 4 m. After removing the surface vegetation and fallen leaves were removed, then employed a uniform sampling method was used to design 17 points for soil sample collection at each site (refer to Fig 1 for the sampling point design). A soil drilling with a diameter of 3 cm was used to extract samples from the 0–20 cm layer. We combined the collected soil samples from each site into a single sample, placed it in self-sealing bags, immediately labeled it, and then transported it back to the laboratory. The samples were then passed through a 2 mm sieve, divided into two portions, and stored in a 4°C refrigerator. Notably, the sampling tools and bags we used were all disinfected with ultraviolet light before sampling. The collected samples were placed in an ice box and transported to the laboratory within 2 hours. We determined the quantity of the soil microbial group and the concentration of soil nitrate nitrogen (NO_3_^-^-N), ammonium nitrogen (NH_4_^+^-N), available phosphorus (AP) and available potassium (AK) within two weeks. We naturally air-dried the second portion to determine the soil’s other physical and chemical properties of the soil.

### 2.3 Measurement indicators and methods

1) **Soil nutrient determination** The total nitrogen (TN) concentration was measured by a Dumas nitrogen meter [22]; the concentrations of NO_3_^-^-N and NH_4_^+^-N in the soil were measured by ultraviolet spectrophotometry and indophenol blue colorimetry [23]; total phosphorus (TP) in the soil was measured by molybdenum antimony colorimetric; and AP in the soil was measured by the measured by the NaHCO_3_ extraction-molybdenum antimony colorimetric [24]; total potassium (TK) was measured by sodium hydroxide fusion-flame photometry, and AK in the soil was measured by the NH_4_OAc extraction‒flame photometry [25].
2) **Determination of soil microbial community diversity:** The determination of soil microbial community diversity was conducted using the method of color average rate of change analysis [26]. We weighed a 10 g soil sample and added it to a conical flask containing 90 mL of sterile physiological saline. We shook the flask in a shaker for 30 minutes and then allowed it to settle for 10 minutes. We collected the supernatant and used the serial dilution method to dilute it 1000-fold. A volume of 150 μL of the diluted solution was transferred to the wells of an ecological hole plate, and the plate was incubated at a constant temperature of 25°C for 7 days in an incubator. We measured the absorbance values every 24 hours using a full-wavelength scanning microplate reader. The diversity indices measured included the Shannon diversity index (H) [27], Simpson diversity index (D) [28] and McIntosh diversity index (U) [29]. The calculation formulas are as follows:

$$H=\sum P_{i}lnP_{i}$$ (1)

$$D=1-\sum P_{i}^{2}$$ (2)

$$\begin{array}{c} \text{U}=\sqrt{\left(\sum {\text{n}}_{\text{i}}^{2}\right)} \end{array}$$ (3)

Where, Pi represents the ratio of the relative absorbance value of the ith well to the total relative absorbance value of the entire plate, and ni represents the relative absorbance value of the ith well.

3) **Soil microbial quantity determination:** The bacteria, fungi, actinomycetes, nitrogen-fixing bacteria, potassium-solubilizing bacteria and phosphate-solubilizing bacteria in soil were cultured using in beef extract-peptone medium, Martin medium, modified Gause’s No. 1 medium, Ashby medium, Ashby medium, sucrose-silicate medium and calcium phosphate inorganic phosphorus culture medium, respectively [30], at 28°C for 2, 3, 4, 5, 7 and 7 days. The colony-forming units in each group were determined using the plate counting method.

### 2.4 Statistical analysis

The data were checked for normality and homogeneity of variance before analysis. Analysis of variance (One-Way ANOVA: least significant difference (LSD) test) was used to identify differences among the four plant communities of soil microbial communities. Principal component analysis (PCA) was used to assess the impact of the sampling sites on *A. adenophora* and the native plant communityunder different plant invasion conditions. Redundancy analysis (RDA) and linear regression were applied to elucidate the relationships between microbial communities’ soil nutrient and diversity indices of soil microbial community. The Pearson correlation coefficient analyzed the relationship between soil microbes and soil nutrients analyzed the relationship between soil microbes and soil nutrients, and the significance test was 2-tailed. The above statistical analyses, except RDA were performed using SPSS 22.0 software (SPSS Inc., Chicago, IL, USA). RDA was performed in CANOCO 5.0 (Microcomputer Power, Ithaca, NY, USA).

## 3. Results

### 3.1 Differences in the soil nutrient concentration of plant community with different degrees of *A. adenophora* invasion

Under the conditions of HIC, LIC, NGC and NDC treatments, there were significant differences of soil nutrient concentration within the plant community that varied in their degree of *A. adenophora* invasion (F = 110.860, *P* < 0.05). The concentrations of TN, NO_3_^-^-N, NH_4_^+^-N, AP, and AK in soil were significantly higher than those in the LIC, NGC and NDC under the condition of HIC (*P *< 0.05). Compared with those in the HIC and LIC, the TP in the NDC and NGC increased significantly. The TP concentrations in the soils of the four plant communities were ranked as follows: NGC > NDC > LIC > HIC (F = 61.973, *P* < 0.05). The TP concentration in the soil of the invasive community was significantly lower than that in the native community (*P* < 0.05). In the severely invasive plant community, the TP concentration was 38.55% and 36.25% lower than that in the native NDC and NGC plant communities, respectively. However, no significant difference was observed in the TP concentration was detected between the NDC and NGC communities (*P *= 0.329). The TK in the NDC was significantly higher than that in the other three groups. The overall potassium concentration in the soil across four plant communities followed this sequence: NDC > LIC > HIC > NGC (F = 49.629, *P* < 0.05). The graphical analysis of the soil nutrient properties in plant communities with different invasion degrees of *A. adenophora* invasion is shown in Table 2.

**Table 2:** Soil nutrient properties of plant community in different degrees of *A. adenophora* invasion.

Soil nutrient properties	TN (g/ kg)	TP (g/kg)	TK (g/kg)	NO_3_^-^-N (mg/ kg)	NH_4_^ + ^-N (mg/ kg)	AP (mg/ kg)	AK (mg/ kg)
HIC	5.75 ± 0.16a	0.51 ± 0.03c	20.04 ± 1.27c	2.86 ± 0.20a	2.19 ± 0.11a	16.66 ± 1.48a	402.89 ± 33.18a
LIC	5.47 ± 0.07b	0.60 ± 0.01b	26.34 ± 0.17b	2.24 ± 0.11b	1.55 ± 0.03b	4.17 ± 0.21b	193.99 ± 11.52b
NGC	2.38 ± 0.02d	0.83 ± 0.02a	11.58 ± 1.32d	0.37 ± 0.00d	0.68 ± 0.03d	2.41 ± 0.07b	78.27 ± 0.50c
NDC	3.53 ± 0.04c	0.80 ± 0.00a	31.85 ± 1.66a	1.25 ± 0.16c	0.96 ± 0.06c	2.05 ± 0.37b	100.81 ± 3.032c

Note: Values are means ± SDs (n = 5). The lowercase letters (a, b, c, d) represent significant differences at 0.05 level between the various plant communities according to ANOVA. HIC: Heavily invasive community; LIC: Lightly invasive community; NGC: native Gramineae community; NDC: native Dicotyledon community. TN: total nitrogen; TP: total phosphorus; TK: total potassium; AP: available phosphorus; AK: available potassium.

### 3.2 Differences in the soil microbial characteristics of plant community with different degrees of *A. adenophora* invasion

Table 3 showed significant variations in the soil microbial taxa among plant communities with varying degrees of *A. adenophora* invasion (F = 438.036, *P* < 0.05). Under the HIC, the quantities of bacteria, fungi, actinomycetes and potassium-solubilizing bacteria in the soil across four plant community types were ranked as follows: HIC > LIC > NDC > NGC (F = 57.314, *P* < 0.05). The abundances of potassium-fixing bacteria and phosphate-solubilizing bacteria were ranked as follows: HIC > LIC > NGC > NDC (F = 132.286, *P* < 0.05). Potassium-solubilizing bacteria and phosphate-solubilizing bacteria in rhizosphere soil were significantly higher than those in the LIC, NGC and NDC (*P *< 0.05). Under the native (NGC and NDC), the Bacteria, Actinomycetes and potassium-solubilizing bacteria in NGC were significantly increased compared to NDC, whereas those of phosphate-solubilizing bacteria were the opposite (*P *< 0.05).

**Table 3:** Soil microbial characteristics of plant community in different degrees of *A. adenophora* invasion.

Soil microbial characteristics	Bacteria (*×10*^*7*^ *CFU/g*)	Fungi (*×10*^*5*^ *CFU/g*)	Actinomycetes (*×10*^*5*^ *CFU/g*)	Nitrogen-fixing bacteria (*×10*^*5*^ *CFU/g*)	Potassium-solubilizing bacteria (*×10*^*5*^ *CFU/g*)	Phosphate-solubilizing bacteria (*×10*^*7*^ *CFU/g*)
HIC	2.86 ± 0.06a	5.55 ± 0.84a	18.07 ± 0.53a	26.19 ± 0.43a	28.25 ± 0.97a	14.22 ± 0.44a
LIC	1.79 ± 0.01b	3.46 ± 0.22b	14.87 ± 0.06b	19.37 ± 0.26b	17.53 ± 0.15b	2.95 ± 0.15b
NGC	0.94 ± 0.00c	1.20 ± 0.03c	6.71 ± 0.21c	12.66 ± 0.32c	10.53 ± 0.18c	1.62 ± 0.04c
NDC	1.47 ± 0.18b	2.22 ± 0.14bc	14.54 ± 0.22b	12.35 ± 0.75c	16.55 ± 0.33b	0.64 ± 0.07d

Note: Values are means ± SDs. The lowercase letters (a, b, c, d) represent significant differences at 0.05 level between the different plant communities, according to ANOVA. HIC: Heavily invasive community; LIC: Lightly invasive community; NGC: native Gramineae community; NDC: native Dicotyledon community.

### 3.3 Analysis of the soil microbial diversity in plant community with different degrees of *A. adenophora* invasion

**Fig 2:**
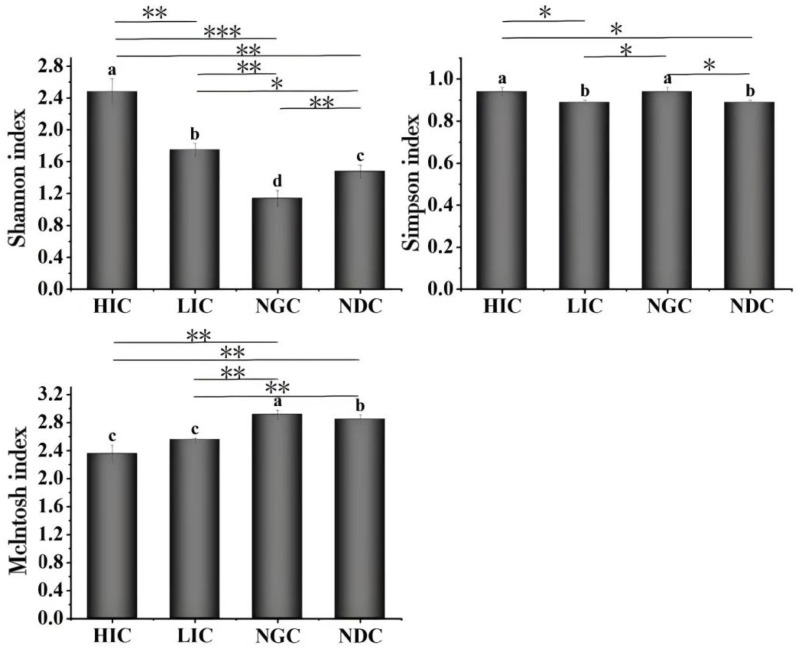
Diversity indices of the soil microbial community between invasive *A. adenophora* and native plant communities. The different small letters represent significant differences at the 0.05 level between the different plant communities. according to ANOVA; The asterisks denote statistically significant differences among the different plant communities according to the t-test (*P < 0.05; **P < 0.01; ***P < 0.001). HIC: Heavily invasive community; LIC: Lightly invasive community; NGC: native Gramineae community; NDC: native Dicotyledon community.

*A. adenophora* demonstrated notable variations in soil microbial diversity across the community with differing degrees of invasion (F = 36.202, *P *< 0.01), as illustrated in Fig 2. The Shannon index of the soil microbes in four plant communities were ranked as follows: HIC > LIC > NDC > NGC (F = 80.302, *P* < 0.01). Under the HIC and NGC, the Simpson index increased compared to LIC and NGC (*P *< 0.05), with no significant difference between the HIC and NGC (*P *> 0.05). In addition, the Mclntosh index in the native groups (NGC and NDC) was significantly higher than the invasive groups (HIC and LIC), with no significant difference between the HIC and LIC (*P *< 0.05). Furthermore, the McIntosh index had significant differences between the HIC and NGC, NDC, andhad significant differences between the HIC and NGC, NDC, and between the LIC and NGC, NDC (*P *< 0.01).

**Fig 3:**
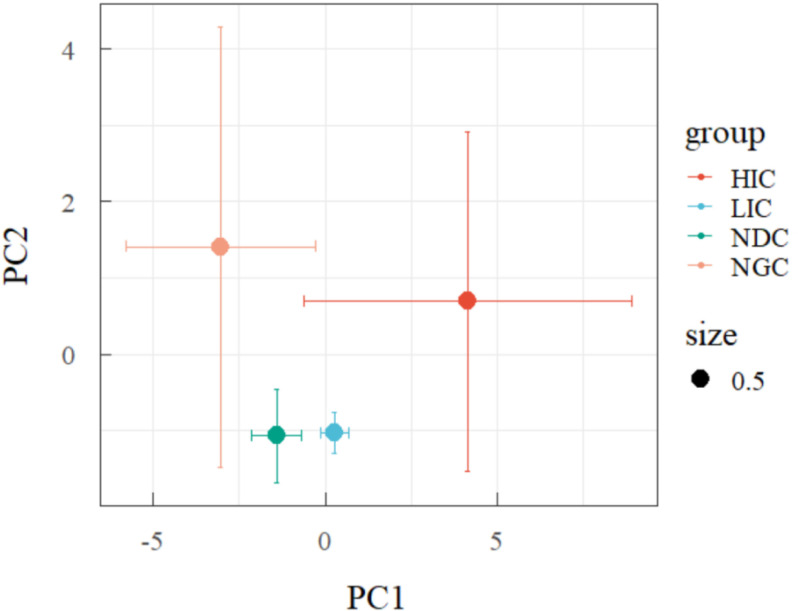
Principal component analysis (PCA) plots demonstrating the effects of sampling site on *A. adenophora* and native plant communities under different plant invasion.

The principal component analysis (PCA) plot showed that the variation of *A. adenophora* and its native plant community (Fig 3). All investigated communities were divided into four groups along the PCA axis 1 and axis 2, with the first axis representing the primary factor because the -3 - 4.5 variation of axis 1 was more significantly than the -0.9 - 1.4 variations of the axis. The observed regression of the HIC substantially differs from that obtained from the NGC. Specifically, the NGC group exhibited the highest value along the PC2, indicating significant differences in its characteristics. On the other hand, the HIC group showed the highest values among the PC1, suggesting distinct differences in its attributes compared to the different groups. Interestingly, the LIC and NDC groups displayed similar patterns of variation, implying a shared similarity in their characteristics.

### 3.4 Analysis of the correlation between soil nutrients and microbes in plant communities with different degrees of *A. adenophora* invasion

**Fig 4:**
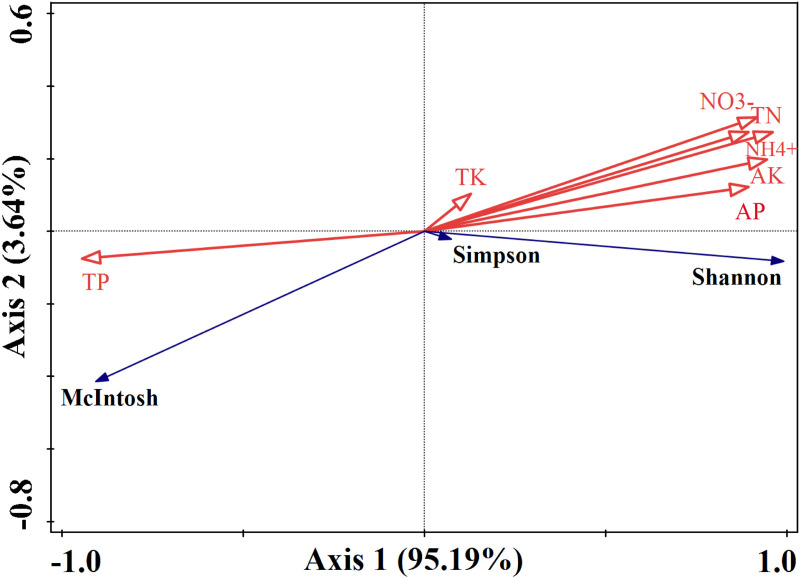
Redundancy analysis (RDA) between the soil nutrient properties and microbial characteristics in invasive *A. adenophora* and native plant communities. The direction of the arrow signifies the positive (right) or negative (left) relationship between environmental variables and species composition. Meanwhile, the length of the arrow represents the strength of the explanatory power of the environmental variable on species composition, with longer arrows indicating a more significant influence.

**Fig 5:**
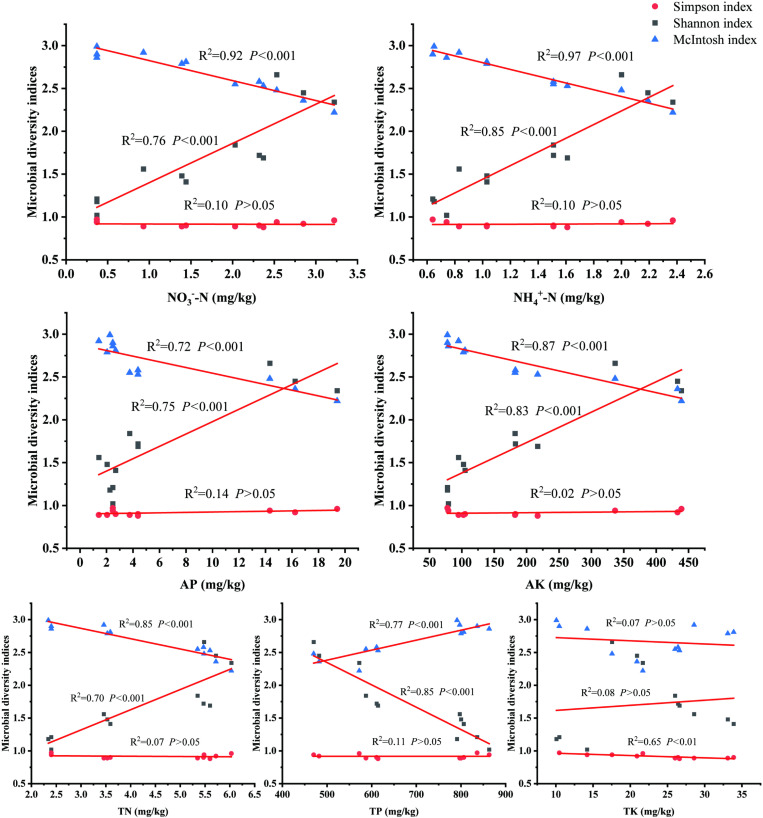
Correlation between rhizosphere soil nutrient properties and soil microbial characteristics in invasive species *A. adenophora* and native plant communities.

The RDA results showed that the first two axes explained 98.83% of the variations in the Shannon index, Simpson index and McIntosh index (Axis 1, 95.19%; Axis 2, 3.64%) in Fig 4. RDA Axis 1, which was highly correlated with the Shannon index, with NO_3_^-^-N, NH_4_^+^-N, AP, AK, TN and TK located along the positive direction, indicating a strong positive contribution and suggesting higher diversity. Meanwhile, RDA Axis 2, which was highly correlated with the McIntosh index, with TP positioned closer, exhibiting a more even distribution. Fig 5 illustrates the soil nutrients and microbes within *A. adenophora* community, which exhibit varying degrees of invasion, demonstrate a corresponding correlation. The NO_3_^-^-N, NH_4_^+^-N, AP, AK, TN, TP and TK affected the Shannon index, Simpson index and McIntosh index. Moreover, the regression results confirmed significant positive correlations between Shannon index and NO_3_^-^-N, NH_4_^+^-N, AP, AK and TN (*P < *0.001), while the significant negative correlations between McIntosh index and NO_3_^-^-N, NH_4_^+^-N, AP, AK and TN (*P < *0.001). However, there was no significant correlation between Simpson index and NO_3_^-^-N, NH_4_^+^-N, AP, AK, TN and TP (*P > *0.05).

## 4. Discussion

### 4.1 Impact of *A. adenophora* invasion and native plant on soil nutrients and its feedback to invasiveness

A significant invasion method is invading exotic plants that surpass native species by modifying rhizosphere soil fertility in communities, promoting their own growth. Studies have discovered that invasive plants change soil nutrient cycling and output in soil ecosystems [31,32] through the decomposition of litter and the release of root exudates, affecting oil nutrient levels and the competition dynamics between native and invasive species [26,33,34]. For example, the invasion of species such as *Solidago canadensis* [35,36], *Berberis thunbergii* [37] and *Melinis minutiflora* [38] increased nitrogen availability in the soil, enhanced the competitive advantage and led to a decline in the diversity of native plant community in invaded habitats. *A. adenophora* was more positively affected by the soil community associated with the native community than by resident natives. Once *A. adenophora* was established, it significantly modified the soil community, enhancing conditions that favored its own growth while suppressing native plant growth, thereby facilitating further invasion [18]. The preliminary study on the invasion impact of the invasive plant *A. adenophora* revealed that it significantly increased soil nitrogen nutrients while decreased phosphorus nutrients in the invaded habitat, which in turn promoted its own growth [39]. Du et al. [40] found that *A. adenophora* enhanced its competitive advantage over native plants by accumulating specific species of *Bacillus*, such as *Bacillus idriensis* [41], *Bacillus toyonensis* [41] and *Bacillus cereus* [42] in its rhizosphere. This accumulation led to a significant increase in the concentrations of NO_3_^-^-N, TN and TC in the soil.

This study showed that as the invasion degree of *A. adenophora* increased, the TN concentration in the soil of the plant community significantly increased. In contrast, the TP concentration exhibited an opposite trend. Additionally, the TK concentration in the *A. adenophora* plant community was between the NGC and NDC. The concentrations of NH_4_^+^-N, NO_3_^-^-N, AP and AK in the soil of the HIC were higher than that in the LIC and the native plant community, and the content in the native Gramineae plant community was the lowest. With the invasion of *A. adenophora*, it can be seen that the concentrations of soil TN, NH_4_^+^-N, NO_3_^-^-N, AP and AK in the plant community increased significantly, which is consistent with the results of previous studies [39]. Still, there are some differences in the concentrations of soil TP and TK [43]. We believe this may be related to the absorption and accumulation of phosphorus and potassium nutrients by *A. adenophora* and other functional plants. Therefore, we believe that *A. adenophora’*s alteration of soil nutrient characteristics in association with different native plants plays a critical role in its successful invasion, with these changes conferring a competitive advantage over native species.

### 4.2 Impact of *A. adenophora* invasion and native plant on soil microbes and its feedback to invasiveness

Interaction between invasive plant species and soil microbial communities is one of the primary factors for the plant invasive ability and susceptibility of receptive communities. The invasion of exotic plants can significantly affect the composition and diversity of soil microbial communities in the invaded habitats, further changing the soil nutrients and affecting the evolution of the communities [44,45] [47]. Some studies indicated that *A. adenophora* changed soil microbial communities, especially the soil nutrition cycling related to microbial groups, and created a favorable soil environment to benefit itself [46]. Kong et al. [47] found that the invasion of *A. adenophora* can lead to changes in the bacterial community structure in the soil of the invasive plant community, and there is a strong correlation between the change of its soil bacterial community structure and the concentration of soil NO_3_^-^-N. Xia et al. [48] compared the rhizosphere microbial composition and functional characteristics of *A. adenophora* and native plant communities found that there were significant differences in the composition and diversity of rhizosphere soil bacteria and fungi in plant communities and their activities, which were considered to be the key for the invasion and colonization of *A. adenophora*.

In this study, the results showed that with the increase of the invasion degree of *A. adenophora*, the richness index and the dominance of the soil microbes in its community were increasing, while the Mclntosh index was decreasing, indicating that the invasion of *A. adenophora* changed the diversity structure of soil microbes in the invaded habitats; In contrast, the dominance index of the soil microbes was higher in the NGC, indicating that a few microbial taxa dominated the community. This pattern may be attributed to the greater abundance of certain soil microbes within this plant community. Furthermore, as the invasion intensity of *A. adenophora* escalated, the overall population of soil microbes within its community experienced a substantial increase, particularly in the quantities of soil bacteria, fungi, actinomycetes, nitrogen-fixing bacteria, potassium-solubilizing bacteria and phosphate-solubilizing bacteria, with the highest counts observed in bacteria and phosphate-solubilizing bacteria. increases the total number of soil microbes in the invasive plant community during the invasion process and changes the composition structure of its soil microbes. Some similar studies have found that *A. adenophora* invasion alters underground microbial communities in invaded areas and modifies soil biota to facilitate its continued invasion [43,49]. Invasion of *A. adenophora* can lead to the accumulation of specific Bacillus taxa in the rhizospheric soil, which in turn can increase the competitive advantage of *A. adenophora* [40], a self-reinforcing invasion mechanism.

### 4.3 The underground competitive relationship between *A. adenophora* invasion and native plants and its ecological effects

Invasive exotic plants show different invasiveness in different ecosystems, and conversely, ecosystem heterogeneity directly affects the invasion and expansion of exotic species. The belowground competitive relationship between invasive and native plants can reveal the invasion mechanism of exotic invasive plants, and it is also an effective way to discover the resistance of native plant communities to invasive species. Invasive exotic plants can affect the soil microbes and soil nutrient levels of the invasive plant community and may affect their interspecific competition and interaction [50,51]. Research showed that *A. adenophora* was more positively impacted by the soil community associated with native communities than resident natives. Once established, it further altered the soil nutrients and soil microbes community in a way that favored itself and inhibited natives, which promoted further invasion [18]. Zhai and Liang [52] showed that *A. adenophora* can change the relationship between soil nitrogen and microbes, and then strengthen the assimilation and transformation of soil nitrogen by soil microbes, which may be an essential mechanism for the success of plants. Moreover, the abundant antagonistic bacteria around the *A. adenophora* rhizosphere tended to resist harmful soil-borne diseases and escape natural enemies [53]. However, some studies have found that various native plant functional groups, such as annual and perennial herbs, weeds and their mixed communities, show different capabilities in influencing the invasion of *A. adenophora.* For example, Jiang et al. [54] demonstrated that under high mixed planting density, *Setaria sphacelata* significantly inhibited the growth of *A. adenophora* (with a 70.1% reduction in individual biomass) and through its aboveground competitive advantage, formed a dense cover that effectively replaced *A. adenophora*, preventing seed germination and re-colonization. The results of soil enzyme activity and nutrient availability in single species and mixed-species communities of *A. adenophora* and *S. sphacelata* revealed that *S. sphacelata* was more effective than *A. adenophora* in activating soil nitrogen, phosphorus and potassium compounds, demonstrating its strong competitive ability against *A. adenophora* [55]. Therefore, restoration and protection of native plant diversity is a practical approach to resist *A. adenophora* invasion.

In this study, the results showed that there was a significant positive correlation between soil nutrients and microbial richness in both the *A. adenophora* community and the native plant community. At the same time, there was a significant negative correlation between soil nutrients and soil microbial evenness, indicating that all plants may consistently impact soil nutrients and soil microorganisms. However, as the invasion of *A. adenophora* increased, the number of soil microbes in the *A. adenophora* community significantly increased, which indicated that the impact of *A. adenophora* on the soil of the invaded habitat was more intense than that of the native plants. Meanwhile, the microbial communities in native plant soils were disrupted, leading to altered soil nutrient cycling and other ecological processes, ultimately giving *A. adenophora* a competitive advantage in the ecosystem. This could suggest that the successful invasion of *A. adenophora* is associated with the suppression of native plant growth or a decline in their competitive abilities, thereby facilitating further invasion. Based on this mechanism, we propose that selecting ideal native plant species (such as those with a stronger ability to change soil microbes and soil nutrients compared to *A. adenophora*) or establishing multi-species native plant groups could be effective strategies. By structuring these plant communities appropriately, we can promote ecosystem stability, providing a crucial approach for the ecological control and resistance of invasive species like *A. adenophora*.

## 5. Conclusions

This study explored the ecological mechanisms of *A. adenophora* invasion by analyzing soil nutrient and microbial differences between the NGC and NDC at different invasion degrees. The results showed that the invasion of *A. adenophora* significantly altered the soil nutrient distribution patterns. Compared to native plant communities, the invaded areas exhibited higher concentrations of TN, NO_3_^-^-N, NH_4_^+^-N, AP and AK, while concentrations of TP and TK were lower. As the invasion level of *A. adenophora* intensified, there was a significant increase in the abundance of bacteria, fungi, actinomycetes, nitrogen-fixing bacteria, potassium-solubilizing bacteria and phosphate-solubilizing bacteria in the soil community. Moreover, the Shannon and Simpson index of microbial diversity were significantly higher in the invaded soil, while the McIntosh index decreased.

Notably, the invasion of *A. adenophora* utilized a “soil nutrient-microbe” synergistic regulation mechanism to maximize resource use efficiency, which which may have been the key mechanism behind its successful invasion. To suppress the spread of *A. adenophora*, it was imperative to develop ecological control strategies based on soil nutrient-microbe interactions. Research findings not only provided important scientific evidence for the prevention and management of *A. adenophora* but also offered a reference paradigm for the design of ecological control strategies for other invasive plants.

## Supporting information

S1 Data Soil nutrient concentration and microbial community characteristics under different levels of *Ageratina* adenophora invasion. (XLSX)

## References

[1] Wan FH, Zheng XB, Guo JY. Biology and management of invasive alien species in agriculture and forestry. Beijing: Science Press; 2005.

[2] Urcelay C, Austin AT. Exotic plants get a little help from their friends. Science. 2020;368(6494):934–6. doi: 10.1126/science.abc3587 32467374

[3] Milbau A, Nijs I, De Raedemaecker F, Reheul D, De Cauwer B. Invasion in grassland gaps: the role of neighbourhood richness, light availability and species complementarity during two successive years. Funct. Ecol. 2005;19(1):27–37. doi: 10.1111/j.0269-8463.2005.00939.x

[4] Román JFC, Hernández-Lambraño RE, Rodríguez de la Cruz D, Sánchez Agudo JÁ. Analysis of the Adaptative Strategy of *Cirsium vulgare* (Savi) Ten. in the colonization of new territories. Sustainability. 2021;13(4):2384. doi: 10.3390/su13042384

[5] Byun C, de Blois S, Brisson J. Plant functional group identity and diversity determine biotic resistance to invasion by an exotic grass. J. Ecol. 2012;101(1):128–39. doi: 10.1111/1365-2745.12016

[6] Gos P, Loucougaray G, Colace MP, Arnoldi C, Gaucherand S, Dumazel D, et al. Relative contribution of soil, management and traits to co-variations of multiple ecosystem properties in grasslands. Oecologia. 2016;180(4):1001–13. doi: 10.1007/s00442-016-3551-3 26830292

[7] Wang CY, Wu BD, Jiang K, Zhou JW, Liu J, Lv YN. Canada goldenrod invasion cause significant shifts in the taxonomic diversity and community stability of plant communities in heterogeneous landscapes in urban ecosystems in East China. Ecol. Eng. 2019;127:504–9. doi: 10.1016/j.ecoleng.2018.10.002

[8] Lamb EG, Kennedy N, Siciliano SD. Effects of plant species richness and evenness on soil microbial community diversity and function. Plant Soil. 2010;338(1–2):483–95. doi: 10.1007/s11104-010-0560-6

[9] Chen FL, Zheng H, Ouyang ZY, Zhang K, Tu NM. Responses of microbial community structure to the leaf litter composition. Acta Pedol Sin. 2011;48(3):603–11.

[10] Li S, Xie D, Ge X, Dong W, Luan J. Altered diversity and functioning of soil and root-associated microbiomes by an invasive native plant. Plant Soil. 2022;473(1–2):235–49. doi: 10.1007/s11104-022-05338-z

[11] Elgersma KJ, Ehrenfeld JG, Yu S, Vor T. Legacy effects overwhelm the short-term effects of exotic plant invasion and restoration on soil microbial community structure, enzyme activities, and nitrogen cycling. Oecologia. 2011;167(3):733–45. doi: 10.1007/s00442-011-2022-0 21618010

[12] Buzhdygan OY, Rudenko SS, Kazanci C, Patten BC. Effect of invasive black locust (Robinia pseudoacacia L.) on nitrogen cycle in floodplain ecosystem. Ecol. Model. 2016;319:170–7. doi: 10.1016/j.ecolmodel.2015.07.025

[13] Qiang S. The history and status of the study on crofton weed (*Eupatorium adenophorum* Spreng.), a worst worldwide weed. J Wuhan Bot Res. 1998;16:366–72.

[14] Wan FH, Guo JY, Wang DH. Alien invasive species in China: their damages and management strategies. Biodivers Sci. 2002;10(1):119–25. doi: 10.17520/biods.2002014

[15] Zhao GJ, Ma YP. Investigation on distribution and damage of *Eupatorium adenophorum* in Yunnan province. J Weed Sci. 1989;3(2):37–40.

[16] Wang R, Wang YZ. Invasion dynamics and potential spread of the invasive alien plant species *Ageratina adenophora* (Asteraceae) in China. Divers. Distrib. 2006;12(4):397–408. doi: 10.1111/j.1366-9516.2006.00250.x

[17] Lu ZJ, Ma KP. Spread of the exotic croftonweed (*Eupatorium adenophorum*) across southwest China along roads and streams. Weed Science. 2006;54(6):1068–72. doi: 10.1614/ws-06-040r1.1

[18] Wan FH, Liu WX, Guo JY, Qiang S, Li BP, Wang JJ, et al. Invasive mechanism and control strategy of *Ageratina adenophora* (Sprengel). Sci China Life Sci. 2010;53(11):1291–8. doi: 10.1007/s11427-010-4080-7 21046320

[19] Gui FR, Yu L, Li YG, Liu WX, Peng H. Effect of mixed-planted *Setaria sphacelata* on rhizophere soil microbes of *Ageratina adenophora*. China Plant Protect. 2010;30(4):10–4. doi: 10.3969/j.issn.1672-6820.2010.04.002

[20] Yang GQ, Wan FH, Liu WX, Guo JY. Influence of two allelochemicals from *Ageratina adenophora* sprengel on aba, iaa and zr contents in roots of upland rice seedlings. Allelopathy J. 2008;21(2):253–62.

[21] Jiang ZL. Ecophysiological mechanisms of competition between invasive weed *Ageratina adenophora* (Asteraceae) and non-invasive herbaceous plants. Beijing: Chinese Academy of Agricultural Sciences; 2007. p.107.

[22] Dumas JBA. Procédés de l’analyse organique. Ann Chim Phys. 1831;47:198–213.

[23] She WW, Bai YX, Zhang YQ, Qin SG, Feng W, Sun YF, et al. Resource availability drives responses of soil microbial communities to short-term precipitation and nitrogen addition in a desert Shrubland. Front Microbiol. 2018;9:186. doi: 10.3389/fmicb.2018.00186 29479346 PMC5811472

[24] Xu GJ, Kang XM, Li W, Li Y, Chai YY, Wu SY, et al. Different grassland managements significantly change carbon fluxes in an alpine meadow. Front Plant Sci. 2022;13:1000558. doi: 10.3389/fpls.2022.1000558 36311073 PMC9606693

[25] Bao S. Soil agrochemical analysis. 3rd ed. Beijing, China: Agricultural Press; 2002. p. 13–28.

[26] Zhang P, Li B, Wu JH, Hu SJ. Invasive plants differentially affect soil biota through litter and rhizosphere pathways: a meta-analysis. Ecol Lett. 2019;22(1):200–10. doi: 10.1111/ele.13181 30460738

[27] Hill MO. Diversity and evenness: a unifying notation and its consequences. Ecology. 1973;54(2):427–32. doi: 10.2307/1934352

[28] Simpson EH. Measurement of diversity. Nat. 1949;163(4148):688.

[29] Németh I, Molnár S, Vaszita E, Molnár M. The Biolog EcoPlate™ technique for assessing the effect of metal oxide nanoparticles on freshwater microbial communities. Nanomaterials (Basel). 2021;11(7):1777. doi: 10.3390/nano11071777 34361164 PMC8308119

[30] Li WH, Zhang CB, Gao GY, Zan QJ, Yang ZY. Relationship between *Mikania micrantha* invasion and soil microbial biomass, respiration and functional diversity. Plant Soil. 2007;296(1–2):197–207. doi: 10.1007/s11104-007-9310-9

[31] Vogelsang KM, Bever JD. Mycorrhizal densities decline in association with nonnative plants and contribute to plant invasion. Ecology. 2009;90(2):399–407. doi: 10.1890/07-2144.1 19323224

[32] Legay N, Clément JC, Grassein F, Lavorel S, Lemauviel-Lavenant S, Personeni E, et al. Plant growth drives soil nitrogen cycling and N-related microbial activity through changing root traits. Fungal Ecol. 2020;44:100910. doi: 10.1016/j.funeco.2019.100910

[33] Liu WX, Niu HB, Wan FH, Liu B. Effects of leachates of the invasive plant, *Ageratina adenophora* (Sprengel) on soil microbial community. Acta Ecologica Sinica. 2010;30(4):196–200. doi: 10.1016/j.chnaes.2010.06.002

[34] Reinhold-Hurek B, Bünger W, Burbano CS, Sabale M, Hurek T. Roots shaping their microbiome: global hotspots for microbial activity. Annu Rev Phytopathol. 2015;53:403–24. doi: 10.1146/annurev-phyto-082712-102342 26243728

[35] Zubek S, Majewska ML, Kapusta P, Stefanowicz AM, Błaszkowski J, Rożek K, et al. Solidago canadensis invasion in abandoned arable fields induces minor changes in soil properties and does not affect the performance of subsequent crops. Land Degrad Dev. 2020;31(3):334–45. doi: 10.1002/ldr.3452

[36] Lu JZ, Qiu W, Chen JK, Li B. Impact of invasive species on soil properties: Canadian goldenrod (*Solidago Canadensis*) as a case study. Biodivers. Sci. 2005;13(4):347. doi: 10.1360/biodiv.050071

[37] Kourtev PS, Ehrenfeld JG, Häggblom M. Experimental analysis of the effect of exotic and native plant species on the structure and function of soil microbial communities. Soil Biol Biochem. 2003;35(7):895–905. doi: 10.1016/s0038-0717(03)00120-2

[38] Kourtev PS, Huang WZ, Ehrenfeld JG. Differences in earthworm densities and nitrogen dynamics in soils under exotic and native plant species. Biol. Invasions. 1999;1(2/3):237–45. doi: 10.1023/a:1010048909563

[39] Dai L, Li HN, Jiang ZL, Wan FH, Liu WX. Invasive effects of *Ageratina adenophora* (Asteraceae) on the changes of effective functional bacteria, enzyme activity and fertility in rhizosphere soil ecosystem. J Ecol Environ Sci. 2012;21(2):237–42. doi: 10.3969/j.issn.1674-5906.2012.02.007

[40] Du EW, Chen YP, Li YH, Sun ZX, Gui FR. Rhizospheric Bacillus-Facilitated effects on the growth and competitive ability of the invasive plant *Ageratina adenophora*. Front Plant Sci. 2022;13:882255. doi: 10.3389/fpls.2022.882255 35774817 PMC9237563

[41] Okyere SK, Wen J, Cui Y, Xie L, Gao P, Zhang M, et al. Bacillus toyonensis SAU-19 and SAU-20 isolated from ageratina adenophora alleviates the intestinal structure and integrity damage associated with gut dysbiosis in mice fed high fat diet. Front Microbiol. 2022;13:820236. doi: 10.3389/fmicb.2022.820236 35250935 PMC8891614

[42] Sun YY, Zhang QX, Zhao YP, Diao YH, Gui FR, Yang GQ. Beneficial rhizobacterium provides positive plant–soil feedback effects to *Ageratina adenophora*. J. Integr. Agric. 2021;20(5):1327–35. doi: 10.1016/s2095-3119(20)63234-8

[43] Li HN, Liu WX, Dai L, Wan FH, Cao YY. Invasive impacts of *Ageratina adenophora* (Asteraceae) on the changes of microbial community structure, enzyme activity and fertility in soil ecosystem. Chin Agric Sci. 2009;42(11):3964–71.

[44] Qi XX, Zhang SY, Lin F, Zhang LL, Yang DL, Huangfu CH, et al. Effect of *Flaveria bidentis* invasion on plant community and soil microbial community of different invaded soil. Acta Ecologica Sinica. 2019;39(22). doi: 10.5846/stxb201901150123

[45] Chauhan P, Sharma N, Tapwal A, Kumar A, Verma GS, Meena M, et al. Soil microbiome: diversity, benefits and interactions with plants. Sustainability. 2023;15(19):14643. doi: 10.3390/su151914643

[46] Kumar M, Kumar S, Verma AK, Joshi RK, Garkoti SC. Invasion of Lantana camara and *Ageratina adenophora* alters the soil physico-chemical characteristics and microbial biomass of chir pine forests in the central Himalaya, India. Catena. 2021;207:105624. doi: 10.1016/j.catena.2021.105624

[47] Kong YH, Kong J, Wang DK, Huang HP, Geng KY, Wang YX, et al. Effect of *Ageratina adenophora* invasion on the composition and diversity of soil microbiome. J Gen Appl Microbiol. 2017;63(2):114–21. doi: 10.2323/jgam.2016.08.002 28239038

[48] Xia Y, Dong MH, Yu L, Kong LD, Seviour R, Kong Y. Compositional and functional profiling of the rhizosphere microbiomes of the invasive weed *Ageratina adenophora* and native plants. PeerJ. 2021;9:e10844. doi: 10.7717/peerj.10844 33717679 PMC7937340

[49] Niu HB, Liu WX, Wan FH, Liu B. An invasive aster (*Ageratina adenophora*) invades and dominates forest understories in China: altered soil microbial communities facilitate the invader and inhibit natives. Plant Soil. 2007;294(1–2):73–85. doi: 10.1007/s11104-007-9230-8

[50] Zhang Z, Dai YY, Wang YF, Lu DL, Wang R. Review of the use of native plants as a control mechanism of invasive weeds. J Biosaf. 2018;27(3):178–85. doi: 10.3969/j.issn.2095-1787.2018.03.004

[51] Peng RM, Peng Y, Xiang GH, Yang ZL. Study on rhizosphere soil nutrients, enzyme activities and microbiological characteristics of different invasive plants. Jiangsu Agric Sci. 2021;49(21):217–23.

[52] Zhai YJ, Liang PF. Effects of alien plant invasion on soil nitrogen and its metabolic enzyme activities of native plants. Bull Soil Water Conserv. 2021;41(6):29–33. doi: 10.13961/j.cnki.stbctb.2021.06.005

[53] Niu HB, Liu WX, Wan FH, Liu B. Screening, identification, and antagonism assessment, of dominant bacteria in *Ageratina adenophora* Sprengel rhizosphere soil. Ying Yong Sheng Tai Xue Bao. 2007;18(12):2795–800. 18333457

[54] Jiang ZL, Liu WX, Wan FH, Li ZY. Competitive effects between *Ageratina adenophora* (Asteraceae) and Setaria sphacelata (Gramineae). Scientia Agricultura Sinica. 2008;41(5):1347–54. doi: 10.3864/j.issn.0578-1752.2008.05.012

[55] Jiang ZL, Liu WX, Wan FH, Li ZY. Comparative studies on seasonal dynamics of soil enzymatic activities and soil nutrient availability in mono- and mixed-culture plant communities of ageratina adenophora and setaria sphacelata. J Plant Ecol. 2008;32(4):900–7. doi: 10.3773/j.issn.1005-264x.2008.04.019

